# Genome-wide patterns of carbon and nitrogen regulation of gene expression validate the combined carbon and nitrogen (CN)-signaling hypothesis in plants

**DOI:** 10.1186/gb-2004-5-11-r91

**Published:** 2004-10-29

**Authors:** Peter M Palenchar, Andrei Kouranov, Laurence V Lejay, Gloria M Coruzzi

**Affiliations:** 1 Department of Chemistry, Rutgers University, Camden, NJ 10003, USA; 2 Center for Bioinformatics, University of Pennsylvania, 423 Guardian Drive, Philadelphia, PA 19104, USA; 3 Laboratoire de Biochimie et physiologie moleculaire des plantes, 2 Place Viala, 34060 Montpellier Cedex 1, France; 4 Department of Biology, New York University, 100 Washington Square East, New York, NY 10003, USA

## Abstract

**Background:**

Carbon and nitrogen are two signals that influence plant growth and development. It is known that carbon- and nitrogen-signaling pathways influence one another to affect gene expression, but little is known about which genes are regulated by interactions between carbon and nitrogen signaling or the mechanisms by which the different pathways interact.

**Results:**

Microarray analysis was used to study global changes in mRNA levels due to carbon and nitrogen in *Arabidopsis thaliana*. An informatic analysis using InterAct Class enabled us to classify genes on the basis of their responses to carbon or nitrogen treatments. This analysis provides *in vivo *evidence supporting the hypothesis that plants have a carbon/nitrogen (CN)-sensing/regulatory mechanism, as we have identified over 300 genes whose response to combined CN treatment is different from that expected from expression values due to carbon and nitrogen treatments separately. Metabolism, energy and protein synthesis were found to be significantly affected by interactions between carbon and nitrogen signaling. Identified putative *cis*-acting regulatory elements involved in mediating CN-responsive gene expression suggest multiple mechanisms for CN responsiveness. One mechanism invokes the existence of a single CN-responsive *cis *element, while another invokes the existence of *cis *elements that promote nitrogen-responsive gene expression only when present in combination with a carbon-responsive *cis *element.

**Conclusion:**

This study has allowed us to identify genes and processes regulated by interactions between carbon and nitrogen signaling and take a first step in uncovering how carbon- and nitrogen-signaling pathways interact to regulate transcription.

## Abstract 2

Microarray analysis and the 'InterAct class' method were used to study interactions between carbon and nitrogen signaling in *Arabidopsis*.

## Background

Carbon and nitrogen are two major macronutrients required for plant growth and development. Specific carbon and nitrogen metabolites act as signals to regulate the transcription of genes encoding enzymes involved in many essential processes, including photosynthesis, carbon metabolism, nitrogen metabolism, and resource allocation [1-5]. For example, studies have shown that carbon sources (for example, glucose or sucrose) affect the expression of genes involved in nitrogen metabolism, including genes encoding nitrate transporters and nitrate reductase [6,7]. Conversely, nitrogen sources (such as nitrate) have been shown to affect the expression of genes involved in carbon metabolism, including genes encoding PEP carboxylase and ADP-glucose synthase [8]. Responses to carbon and nitrogen result in important changes at the growth/phenotypic level as well. For example, carbon and nitrogen treatments have antagonistic effects on lateral root growth [9], while their effect on cotyledon size, chlorophyll content and endogenous sugar levels appear to be synergistic [10].

In plants, there are multiple carbon-responsive signaling pathways [11-13], and progress has been made in uncovering parts of the sugar-sensing mechanisms in plants, including the identification of a putative glucose sensor, hexokinase [14]. However, our current knowledge of the mechanisms by which genes and biological processes are regulated by carbon signaling in plants and how they are regulated at the level of transcription is still limited. For example, a search of the PlantCare [15,16] and TRANSFAC [17] databases revealed only seven plant *cis *elements that have been shown to be carbon-responsive *cis *elements (C-elements) and none has been identified from studies in *Arabidopsis thaliana*. Although much less is known concerning the mechanisms controlling nitrogen signaling, microarray analysis has been used to identify nitrogen-responsive genes [8,18]. It has recently been proposed that glutamate receptor 1.1 (AtGLR1.1) functions as a regulator of carbon and nitrogen metabolism in *A. thaliana *[19], but a global understanding of the genes and processes that are regulated by carbon and nitrogen signaling in plants and the mechanism by which this occurs is still lacking.

**Figure 1:**
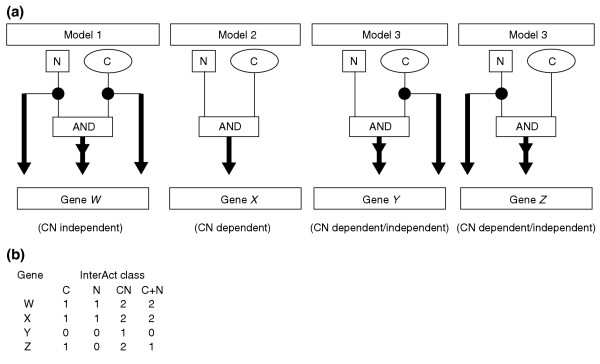
Transcriptional regulation by carbon and nitrogen interactions. **(a) **Interactions between carbon (C) and nitrogen (N) signaling can be explained by three models, and an example(s) of each is given. Model 1, carbon and nitrogen regulation are independent and therefore are additive. Model 2, carbon and nitrogen are dependent, as both are required for an effect. Model 3, there is a dependent and independent component to carbon and nitrogen regulation. Two examples of Model 3 are shown (genes *Y *and *Z*). For gene *Y*, nitrogen only has an effect in the presence of carbon, while for gene *Z*, carbon only has an effect in the presence of nitrogen. **(b) **The assignment of genes *W*, *X*, *Y*, and *Z *to InterAct classes.

Previously, microarrays were used to identify genes and biological processes regulated by interactions between carbon and light signaling in *A. thaliana*, including the identification of a putative *cis *regulatory element that is responsive to either light or carbon signals [13]. In this study, we present a genome-wide analysis of the effects of transient carbon and/or nitrogen treatments on mRNA levels, with a particular focus on genes whose mRNA levels are affected by the carbon and nitrogen (CN) treatment. This study has enabled us to evaluate a number of models for intersections between carbon and nitrogen signaling (Figure 1) and to identify genes and biological processes that are regulated by the interactions between carbon and nitrogen signaling pathways. In addition, we have identified putative *cis *elements that may be responsible for coordinating a gene's responses to both these signaling pathways.

## Results

### Testing models of carbon and nitrogen regulation

The goal of this study was to use a genomic approach to test the hypothesis that carbon and nitrogen signaling pathways interact to regulate the expression of genes in *Arabidopsis*. We predicted six general models that could describe the possible modes of gene regulation due to carbon, nitrogen and CN together. Three of these models do not involve interactions between carbon and nitrogen signaling. The 'No effect' model includes genes not regulated by carbon, nitrogen and/or CN. The 'C-only' model includes genes regulated only by carbon. Finally, the 'N-only' model includes genes regulated only by nitrogen. Three additional models are needed to describe the regulation of genes affected by interactions between carbon and nitrogen signaling (Figure 1a). Model 1 (CN independent) depicts a gene *W*, for which carbon and nitrogen signals act as independent pathways, so that the effects of carbon and nitrogen are additive. Model 2 (CN dependent) depicts a gene *X*, for which regulation requires carbon and nitrogen, and neither carbon alone nor nitrogen alone has an effect. Model 3 (CN dependent/independent) incorporates both an independent and a dependent component to the interactions of carbon and nitrogen signaling. For gene *Y*, carbon alone has an independent inductive effect, while nitrogen has a carbon-dependent effect as it can enhance the effect of carbon, but has no effect on its own (Model 3 CN-enhanced). For gene *Z*, nitrogen alone has an independent inductive effect, while carbon has a nitrogen-dependent effect. These general models can be broken down into more descriptive sub-models. For example, Model 2 can be broken into two sub-models for which CN results in either an inductive or repressive effect.

**Figure 2:**
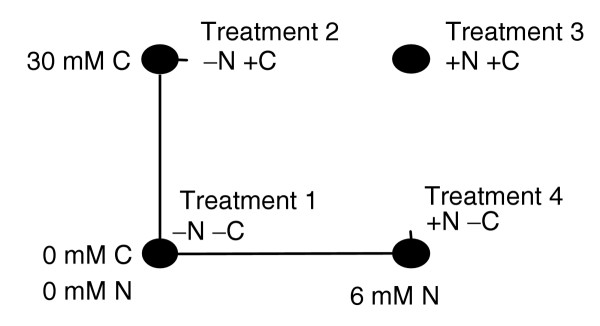
Treatments for carbon and nitrogen interaction studies. +C, -C, with and without carbon, respectively. +N, -N, with and without nitrogen, respectively.

To test the *in vivo *significance of the above models, a microarray analysis of RNA from plants treated transiently with distinct carbon and nitrogen treatments was carried out, and the results were analyzed to determine the carbon and nitrogen regulation of different genes. For this study, we analyzed RNA isolated from *Arabidopsis *seedlings exposed to four different transient carbon and/or nitrogen treatments (-C/-N, +C/-N, -C/+N, and +C/+N) (Figure 2) using Affymetrix whole-genome microarray chips. Analysis of gene expression across these treatments was performed on the whole genome using InterAct Class [13,20], an informatic tool that enabled us to classify genes into each of the above models based on their relative responses to carbon and/or nitrogen treatments. The analysis of the microarray data with InterAct Class enabled us to group genes whose relative responses to carbon, nitrogen and CN were similar to each other. In this case, each InterAct class is made up of four values listed in the following order: value 1 = the expression due to carbon; value 2 = the expression due to nitrogen, value 3 = the expression due to carbon and nitrogen supplied as a combined treatment (CN); and value 4 = the synthetic expression of C+N calculated by adding the expression due to carbon plus the expression due to nitrogen, which is a 'virtual' treatment.

InterAct Class is a ranking system used to qualitatively compare gene-expression profiles across multiple treatments. For each gene, each treatment is assigned a value representing the effect of the treatment on the expression of that gene. Treatments that result in repression of a gene are assigned a negative number, treatments that do not significantly affect a gene are assigned zero, and treatments that cause induction are assigned a positive number. If more than one treatment causes induction or repression, the treatments are ranked so that the treatment that causes the most induction or repression will be assigned the number furthest from zero. The four hypothetical genes in Figure 1a (*W*, *X*, *Y *and *Z*) were classified by InterAct Class (Figure 1b), demonstrating that, with this program it becomes easy to determine whether the regulation of a gene is due to a complex (non-additive) interaction between carbon and nitrogen signaling. For such genes, the value assigned to CN (the third InterAct Class number) will be higher or lower than the value assigned to C+N (the fourth InterAct Class number). These genes will fall into Models 2 and 3 (Figure 1b, genes *X*, *Y *and *Z*).

Out of 23,000 genes on the Affymetrix chip, 3,652 passed our stringent filtering criteria for reproducibility among treatment replicates and were assigned an InterAct class. Our subsequent analysis of the expression patterns of these 3,652 genes validated the existence of 60 different InterAct classes (Table 1 and Additional data file 1). These 60 InterAct classes represent a broad spectrum of expression patterns that validate each of the six general models for gene regulation. This analysis shows that of the 3,652 genes in the analysis, the vast majority (2,485) is responsive to carbon and/or nitrogen treatment. Moreover, almost half of these genes (1,175 genes) are regulated by an interaction between carbon and nitrogen signaling (Table 1). For example, there are 175 genes that are in Model 3 CN-enhanced, for which expression due to CN is greater than expression due to C+N (Table 1 and Additional data file 1). This suggests that an interaction between carbon and nitrogen signaling affects the expression of this set of genes.

### MIPS funcat analysis uncovers biological processes that are regulated by carbon and/or nitrogen

The InterAct classes were assigned to one of the six general models. To identify biological processes that contain a significant number of genes regulated by carbon, nitrogen and/or CN, we determined which Munich Information Center for Protein Sequences (MIPS) functional categories (funcats) [21,22] were statistically under-represented in the No effect model (InterAct class 0000), compared to all the genes assigned an InterAct class (Table 2) (not to all the genes in the genome; this takes into account any bias that may have occurred as a result of the filtering process before InterAct class analysis). Under-representation of a biological process in the No effect model means that for that particular funcat, there are fewer genes in the No effect model than expected on the basis of how all the genes assigned to an InterAct class behave. This means that processes under-represented in the 0000 InterAct class contain a significant number of genes that respond to carbon and/or nitrogen treatments compared to the general population of genes in the analysis.

For example, 31.6% (1,089/3,447) of the genes assigned to an InterAct class and a funcat are assigned to the No effect model (Table 2). This percentage was used as a basis of comparison to determine if genes in any specific funcat varied significantly from the general population. For example, if genes in the metabolism funcat are not regulated by carbon and/or nitrogen in a significant fashion, the number of genes expected to be in the No effect model would be equal to the total number of genes in the metabolism funcat that are assigned an InterAct class (496) times 0.316, which would equal 156.7 genes. However, the actual number of metabolism genes in the No effect model is 120, which is significantly less than 156.7 (*p*-value = 6.0 × 10^-4^). Therefore, the metabolism funcat is under-represented in the No effect model, showing that metabolism displays significant regulation by carbon and/or nitrogen. This analysis revealed several primary funcats (01 = metabolism, 02 = energy and 05 = protein synthesis) that are significantly under-represented in the No effect model (Table 2). Thus, a significant number of genes involved in metabolism, protein synthesis and energy respond to carbon, nitrogen and/or CN.

For the funcats that are under-represented in the No effect model, this type of analysis was extended to examine the regulation of these funcats in all of the sub-models. This analysis enabled us to determine into which sub-models the genes from these funcats fell and to determine whether the genes in these funcats are under- and over-represented (-S and +S respectively) in these sub-models (Table 3) (see Additional data file 1 for the *p*-value, and the funcat analysis extended to every sub-model and every funcat).

### Identification of *cis *elements associated with CN-regulated genes

To begin to elucidate the mechanisms that control gene regulation in response to carbon and nitrogen treatments, we sought to identify putative *cis *elements that might be responsible for regulating genes in Model 3 CN-enhanced (Table 1). These genes are likely to contain *cis *elements involved in interactions between carbon and nitrogen signaling because the expression due to CN is greater than that due to C+N. Previously, genes that are biologically related and similarly expressed were used to find putative *cis*-regulatory elements involved in carbon and/or light regulation [13]. For this study, to identify related genes in metabolism, we added a new statistical functionality to the informatic tool PathExplore [23], which enabled us to identify metabolic pathways that contain more genes than expected in a list of genes [24]. As used here, PathExplore is useful to find functionally related genes from analyses that combine data from multiple microarray chips (for example, InterAct Class and clustering). In this case, we searched for pathways that contained more than the expected number of genes in Model 3 CN-enhanced, compared to the general population. Three genes involved in ferredoxin metabolism were found to be over-represented in Model 3 CN-enhanced (*p*-value = 0.022) (Table 4a). These genes were also found to be induced in roots and shoots of nitrate-treated plants [18], and the protein products of these genes are all predicted to be localized to the chloroplast [25], further suggesting that they are biologically related and co-regulated.

As we found that genes in the funcat protein synthesis are over-represented in Model 3 CN-enhanced (Table 3), we selected a set of genes in protein synthesis that are in Model 3 CN-enhanced for additional *cis *search analysis. Four nuclear genes encoding ribosomal proteins predicted to be localized to the mitochondria [25] were assigned to InterAct class 1021 (Table 4b). These four genes meet the criteria of being biologically related and having similar expression patterns and were also analyzed for potential *cis*-regulatory elements. Over-represented motifs in the promoters of the four protein synthesis genes or the three ferredoxin metabolism genes were identified using AlignAce [26,27] (AlignAce motifs).

**Figure 3:**
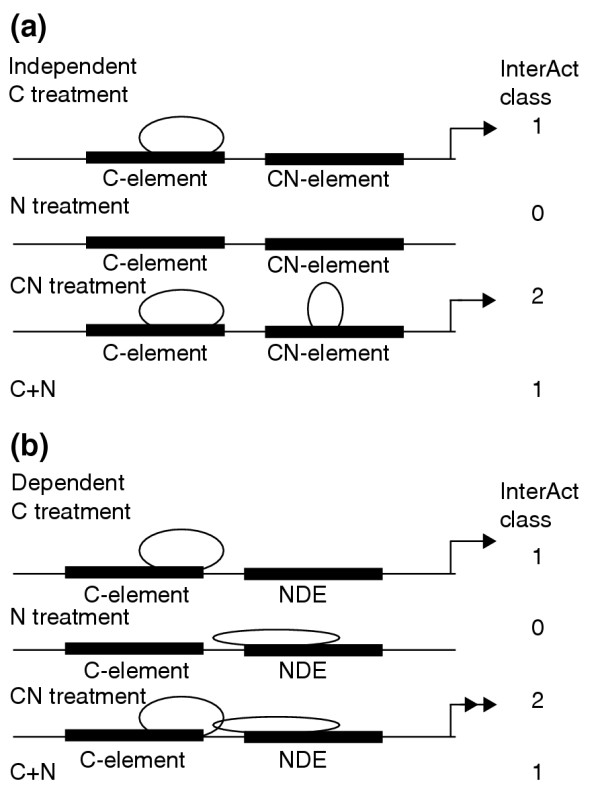
Two general mechanisms that would result in CN expression being greater than C+N. **(a) **Carbon (C) and CN regulatory elements are independent and do not interact. The data do not allow us to rule out the possibility that the C-element is inactive in the presence of CN and that the CN-element alone results in more expression than the C-element. **(b) **CN and carbon regulation are dependent. The increase in expression due to CN requires two interacting *cis *elements, one of which is a C-element and the other a nitrogen-dependent enhancer of carbon regulation (NDE).

We predicted two general mechanisms for which we might be able to identify *cis*-regulatory elements by which carbon and nitrogen can have a non-additive effect (for example, Model 3 CN-enhanced) on the transcription of a gene (Figure 3). These models predict that because the genes used for *cis *discovery are induced by carbon alone, there must be a transcription factor (and cognate *cis *element) that responds to carbon alone. Such carbon-responsive *cis *elements (C-elements) can be identified because they should also be over-represented in the promoters of genes that are induced by carbon alone (the C-only inductive model). From this analysis, a number of the AlignAce motifs identified from the ferredoxin metabolism and protein synthesis genes in the Model 3 CN-enhanced were also shown to be associated with C-only inductive model genes (Table 5; C1-C11). The simplest model that could result in the expression due to CN being greater than C+N is depicted in Figure 3a. In this model, the promoters that contain a C-element are also regulated by a completely independent transcription factor (and cognate *cis *element) that responds specifically to a CN-signaling pathway (Figure 3a). If such a CN-responsive *cis *element (CN-element) exists, it would be predicted to be over-represented in the promoters of genes in Model 3 CN-enhanced, but would not be over-represented in the C-only inductive model. Two of the AlignAce motifs fit this pattern (motifs CN1 and CN2, Table 5), suggesting that they are CN-elements.

If CN1 and CN2 regulate gene expression, they might be expected to be evolutionarily conserved. Unfortunately, *A. thaliana *and/or *Oryza sativa *have multiple genes encoding ferredoxin and ferrodoxin reductase, and as such, the true orthologs of the genes used for this analysis can not be conclusively identified for a promoter analysis (the same is true for the ribosomal genes used for analysis). Another prediction is that if CN1 and CN2 regulate gene expression, biologically related genes might also contain CN1 and CN2. Interestingly, ferredoxin-dependent nitrite reductase (At2g15620) contains three copies of CN1 and one copy of CN2 in its promoter. This gene is in Model 3 CN-enhanced (InterAct class 1021), its protein product is localized to the chloroplast [25] and its expression is induced in shoot and roots of nitrate-treated plants [8], suggesting that the gene is biologically related to and co-regulated with the ferredoxin and ferredoxin reductase genes used for this analysis. We next tested if finding three copies of CN1 and one copy of CN2 in the promoter of ferredoxin-dependent nitrite reductase was statistically likely by testing randomized versions of the promoter. We found that three copies of CN1 were unlikely (*p*-value = 0.0364), but it would not be unlikely to find one copy of CN2 (*p*-value = 0.200). In addition, a total of four copies of CN1 and CN2 was very unlikely (*p*-value = 0.018) in any combination (for example, three CN1 and one CN2, two CN1 and two CN2, or one CN1 and two CN2, and so on).

As *A. thaliana *has only one copy of ferredoxin-dependent nitrite reductase, we searched the *O. sativa *genome sequence for ferredoxin-dependent nitrite reductase genes. Again, we found only one gene [28]. BLAST [29] did not find enough similarity between the promoters of the *A. thaliana *ferredoxin-dependent nitrite reductase gene and the *O. sativa *gene for an alignment. Despite this lack of similarity, we tested for the presence of CN1 and CN2 in the promoter of this gene; three copies of CN1 (*p*-value = 0.052) and one copy of CN2 (*p*-value = 0.389) were found. Again, it was very unlikely that a total of four copies of CN1 and CN2 (*p*-value = 0.045) would occur in the promoter sequence.

### Identification of nitrogen-dependent enhancers of carbon regulation (NDEs)

A second mechanism by which the expression due to CN could be greater than C+N could involve a nitrogen-responsive *cis *element that alone has little or no effect on gene regulation, but when present in combination with a C-element, enhances the induction caused by carbon and is dependent on a carbon-responsive transcription factor (Figure 3b). Other regulatory modules in plants have been identified in which the regulation due to one *cis *element requires the presence of another [30]. In the example examined here, the nitrogen-dependent *cis *element enhances the induction caused by the C-element, making it a nitrogen-dependent enhancer of carbon regulation (NDE). To identify NDEs, our strategy for *cis *element identification was modified. NDEs would be expected to be over-represented in the promoters of Model 3 CN-enhanced genes, but only when present in combination with a separate C-element, as both elements are required to give the enhanced expression due to CN. However, some of the AlignAce motifs are potentially involved in regulating expression due to the carbon treatment in cooperation with the already identified C-elements. These *cis *elements would be similar to NDEs as they would be over-represented in genes induced by carbon in combination with the already identified C-elements. As these motifs are not NDEs, we sought to identify them and remove them from the analysis. AlignAce motifs were tested to determine whether they are over-represented in the promoters of genes whose promoters contain any of the C-elements and are in the C-only inductive model. Those that were found to be over-represented were eliminated from further analysis because these motifs are potentially involved in carbon regulation and are not NDEs. Next, the remaining 33 AlignAce motifs were tested to determine if any are NDEs by determining whether they are over-represented in combination with a C-element within the promoters of the Model 3 CN-enhanced genes. Seven of the potential NDEs are over-represented (*p*-value < 0.05) with at least one C-element in the promoters of the Model 3 CN-enhanced genes, resulting in 12 significant combinations between putative NDEs and C-elements (that is, some of the potential NDEs are over-represented with more than one C-element; data not shown).

To determine if this approach resulted in an enrichment of NDEs, the promoter sequence of each gene was randomized, and the same test was performed. This enabled us to determine whether the remaining 33 AlignAce motifs were over-represented in combination with each C-element in the randomized promoters of the Model 3 CN-enhanced genes. Sets of the randomized promoters (200 sets) were tested, and none of them had as many significant pairs of potential nitrogen-dependent enhancers of carbon regulation and C-elements than the 12 found in the actual promoters. This randomization proves that our approach successfully enriched for NDEs in the actual promoters of the Model 3 CN-enhanced genes and that all the observed significant combinations cannot be due to false positives (*p*-value < 0.005).

Not surprisingly, each of the seven potential NDEs was found to be over-represented with C-elements using the randomized promoters. This shows that false positives can occur in testing for NDEs. The results from the randomized promoters were used to identify which potential NDEs are over-represented with more C-elements than expected (that is, all the combinations for that NDE cannot be explained by false positives). Two NDEs (N1 and N2) were found to be associated with C-elements (Table 5; C3, C6, C7 and C10) in six (N1C6, N1C7, N2C3, N2C6, N2C7 and N2C10) of the 12 significant combinations between the 33 remaining AlignAce motifs and the C-elements. N1 and N2 are involved in more significant combinations than expected on the basis of the randomization study (Table 6; last column).

If N1 or N2 work with the C-elements (C3, C6, C7 and C10) to regulate gene expression in response to CN, then genes that contain both motifs and are in Model 3 CN-enhanced should be misrepresented in certain functional groups as these genes are truly co-regulated. This misrepresentation should occur not only with respect to the genome, but also with respect to the genes in Model 3 CN-enhanced. This result is expected because these genes are more closely related to each other than to the other genes in Model 3 CN-enhanced, and because their CN regulation is the result of the action of the same transcription factor(s). Funcat analysis was used to determine if any functional categories were misrepresented in the genes whose promoters contain N1C6, N1C7, N2C3, N2C6, N2C7 or N2C10 and are in Model 3 CN-enhanced. As the genes used to derive most of the pertinent *cis *motifs encode proteins that are localized to mitochondria, we also tested to see if these genes were misrepresented in the predicted localization of the proteins they encode with respect to the genes in Model 3 CN-enhanced. For the genes whose promoters contain N1C6, N1C7, N2C3, N2C6, N2C7, or N2C10 and are in Model 3 CN-enhanced, only the 'protein synthesis' funcat was found to be misrepresented amongst the primary funcats as compared to all the genes in Model 3 CN-enhanced (Table 7). The genes predicted to encode mitochondria-localized proteins are over-represented for some combinations, but genes localized to the cytoplasm or chloroplast are never misrepresented (Table 7). Two combinations (N2C3 and N2C8) do not show over-representation in protein synthesis and/or genes encoding mitochondria-localized proteins, suggesting they are false positives. All the others show over-representation in some category, further suggesting the potential biological relevance of these *cis *elements (Table 7).

## Discussion

This report contains the one of the first genome-wide investigations of carbon- and nitrogen-signaling interactions in *A. thaliana *[31]. While the focus of our analysis is related to genes controlled by carbon and nitrogen interactions, information from this study can also be used to globally identify genes and processes responsive to regulation by carbon or nitrogen alone. This type of analysis reveals that carbon is a more ubiquitous regulator of the genome compared to nitrogen. The most obvious manifestation of this is the number of genes assigned an InterAct class that are regulated by C-only (1,310) versus N-only (4) (Table 1). This result is not surprising, because carbon plays a major part in many biological processes and is therefore a major regulator of those processes. However, our studies show that nitrogen has a significant role in modifying the effect of carbon on gene expression. In particular, it is noteworthy that many genes show a response to CN (208 genes) treatment that is different from plants treated with carbon alone (Table 1 and Additional data file 1). This analysis demonstrates that nitrogen does have an effect on gene expression, but that in the vast majority of cases, the nitrogen effect is largely carbon-dependent. The carbon dependence of nitrogen regulation may reflect the metabolic interdependence of carbon and nitrogen. For example, carbon skeletons are required on which to assimilate nitrogen into amino acids.

Biological processes containing genes that respond significantly to carbon, nitrogen and/or CN were initially identified by finding MIPS funcats [21,22] that contained genes that were under-represented in InterAct class 0000 (the No effect model) (Table 2). Funcats under-represented in the No effect model have a significant number of genes regulated by carbon and/or nitrogen. It is not surprising that processes like metabolism, protein synthesis, and energy are under-represented in the No effect model. These processes control metabolism or require energy generated by metabolism, and therefore expression of genes involved in these processes are likely to change in response to changes in levels of carbon, nitrogen and/or CN caused by external feeding or depletion after starvation. Protein synthesis regulation might be because it is a downstream process responding to an increase of amino acids as a result of feeding carbon, nitrogen and/or CN.

To gain a better understanding of how the metabolism, energy and protein synthesis funcats are regulated by carbon and/or nitrogen, the sub-models in which they are misrepresented were identified (Table 3). This analysis revealed that the energy funcat is over-represented in InterAct classes that correspond to repression by carbon. It has been shown that carbon sources repress the expression of genes involved in photosynthesis [32]. As photosynthesis genes are part of the energy funcat, the photosynthesis sub-funcat (02.40) was tested and found to be over-represented in the C-only repressive model, in agreement with the previously observed repression of photosynthesis genes by carbon [32].

Surprisingly, metabolism is over-represented in Model 3 CN-suppressed, indicating that many of the genes involved in metabolism show less expression due to CN than expected. The majority of the genes (28 out of 34) were repressed by carbon, induced by nitrogen and repressed by CN, and were assigned to InterAct classes such as -21-2-1 (see Additional data file 1). Several of these genes encode enzymes involved in the catabolism of complex carbohydrates, including β-fructofuranosidase (At1g12240), β-amylase (At3g23920) and β-glucosidase (At3g60130 and At3g60140). *ASN1 *(At3g47340), which has been proposed to be involved in producing asparagine for the transport of nitrogen when carbon levels are low and has been shown to be repressed by carbon [32], was assigned Model 3 CN-suppressed (-21-2-1). In addition, *GDH1 *(At5g18170), which has been proposed to be involved in ammonia assimilation when ammonia levels are high, is repressed by carbon, and induced by nitrogen [33], and was assigned InterAct class -21-2-1, again a Model 3 CN-suppressed class. These genes therefore seem to be regulated as a result of decreased levels of carbon, increased levels of nitrogen or an imbalance between carbon and nitrogen. For example, when carbon sources are limiting (nitrogen is in excess), *ASN1 *is induced because it is involved in shifting the excess nitrogen to asparagine, as asparagine is an efficient way to store and transport nitrogen with respect to carbon [34]. However, when carbon is in excess or carbon and nitrogen are balanced, *ASN1 *is repressed. The regulation of these genes demonstrates the exquisite control of metabolic genes required to balance carbon and nitrogen availability.

Our studies also showed that protein synthesis is one of the processes most affected by the interactions between carbon and nitrogen signaling (Table 3). In addition, the funcat entitled 'protein with binding function or cofactor requirement' (structural or catalytic) is also over-represented in Model 3 CN-enhanced (see Additional data file 1), partly due to genes that encode proteins involved in translation, including At4g10450 (putative ribosomal protein L9 cytosolic; InterAct class 2132) and At4g25740 (putative ribosomal protein S10; InterAct class 1021) (see Additional data file 1). This suggests that protein synthesis is regulated by carbon (see above), but also by complex interactions between carbon and nitrogen signaling.

Little work has been done on the transcriptional control of protein synthesis by carbon and/or nitrogen signaling in plants. However, it has been shown in yeast that genes encoding ribosomal proteins are induced by nitrogen in the presence of carbon; whether this induction by nitrogen requires carbon to be present was not addressed in the yeast study [35]. Furthermore, in the fungus *Trichoderma hamatum*, the gene for ribosomal protein L36 is regulated by interactions between carbon and nitrogen, as it is induced only by CN, and not by carbon or nitrogen alone [36]. Our studies of carbon and nitrogen regulation of gene expression in plants, combined with the studies in fungi, suggest that transcriptional regulation of genes involved in protein synthesis by carbon and nitrogen signaling interactions is evolutionarily conserved.

Finally, we sought to identify the *cis*-regulatory mechanisms involved in carbon and nitrogen signaling interactions. We hypothesized that there could be two general transcriptional mechanisms that would result in the expression due to CN being greater than that due to C+N (Figure 3). In one case, the regulation due to carbon and the regulation due to CN are completely independent (Figure 3a), and in the other case, the regulation due to nitrogen is dependent on a carbon-responsive transcription factor and *cis *element (Figure 4b). Since CN1 and CN2 (Table 5) are over-represented in Model 3 CN-enhanced genes (for example, InterAct class 1021) independently of a C-element, we propose that CN1 and CN2 regulate gene expression due to CN that is independent of a C-element (Figure 3a). This hypothesis is supported because CN1 and CN2 were found in the ferredoxin-related genes, which contain no C-elements that are over-represented in Model 3 CN-enhanced. However, we cannot rule out the possibility that CN1 and CN2 are promiscuous NDEs (Figure 3b) that interact with many C-elements, which might result in over-representation of CN1 and CN2 in Model 3 CN-enhanced genes, but not in over-representation of a specific C-element.

Further analysis suggests that CN1 is involved in regulating the expression of ferredoxin-dependent nitrite reductase. Finding three copies of CN1 in the promoter of the *A. thaliana *ferredoxin-dependent nitrite reductase gene is statistically unlikely (*p*-value = 0.0364), and while three copies in the promoter of the *O. sativa *gene did not reach the 0.05 cutoff, this might represent some small change in the specificity of the regulating factor between *O. sativa *and *A. thaliana*. The failure of BLAST to detect any similarity between the promoters of these two genes suggests that their transcriptional regulators share very little sequence specificity, so a slight change in specificity is not unexpected. The same analysis suggests that CN2 is a false positive because it is not over-represented in the promoters of ferredoxin-dependent nitrite reductase genes. However, we cannot rule out the possibility that the combination of CN1 and CN2 is what is important in regulating these genes, as having a total of four copies of CN1 and CN2 is unlikely in the promoters of both genes. One possibility is that there is a positional relationship between the copies of CN1 and CN2 that is important. From a quick visual inspection, there does not appear to be a conserved relationship between the three copies of CN1 and one copy of CN2 in the *A. thaliana *and *O. sativa *promoters. These issues will have to be resolved by further experimental work; however, these results do suggest that ferredoxin, ferredoxin reductase and ferredoxin-dependent nitrite reductase are co-regulated by carbon and nitrogen due to CN1 and/or CN2. CN1 and/or CN2 therefore might act to link nitrogen reduction and energy metabolism.

Our analysis found CN-elements in the promoters of the ferredoxin-related genes (Table 4a), but not in those of the nuclear-encoded ribosomal mitochondrial protein genes (Table 4b). Also none of the C-elements found in the ferredoxin-related genes (C1 through C5) is over-represented in the Model 3 CN-enhanced genes, suggesting that these elements have no role in CN regulation and that the CN and carbon signaling are independent (Table 5). In contrast, most of the C-elements in the promoters of the ribosomal protein genes are also over-represented in the promoters of the Model 3 CN-enhanced genes (C6 through C9), suggesting that they have a role in carbon and CN regulation. In addition, the majority of the C-elements (C6, C7 and C10) found to be over-represented in combination with NDEs (N1 and N2), and the most statistically significant of these enhancers (N2), was found in the promoters of the ribosomal proteins (Table 6). This suggests that the CN transcriptional regulation of genes for ribosomal proteins is primarily due to NDEs (Figure 3b). Thus, it is not surprising that many of the genes potentially regulated by the combination of C-elements and NDEs are involved in protein synthesis (Table 7). However, the putative NDEs most probably regulate genes involved in a number of different biological processes. For example, genes that contain the combination N1C7 and are in Model 3 CN-enhanced include metabolic genes (for example, At3g25900 (homocysteine S-methyltransferase), At2g30970 (aspartate aminotransferase) and At3g52940 (C-14 sterol reductase)), histone-related proteins (for example, At1g54690 (histone H2A) and At2g27840 (histone deacetylase-related)), and putative signaling/regulatory proteins (for example, At4g39990 (Ras-related GTP-binding protein BG3), At5g38480 (14-3-3 protein) and At3g18130 (guanine nucleotide-binding protein)).

This analysis represents a first step in understanding how carbon and nitrogen signaling interact to control gene expression and has identified genes and putative *cis *elements that are responsive to carbon and nitrogen signaling interactions. It is noteworthy that the putative CN-elements and NDEs represent *cis *elements that have not been previously identified and as such may represent novel components of the CN regulatory circuit. Further study of the identified genes and *cis *elements is required to bring about a complete understanding of interactions between carbon and nitrogen signaling.

## Materials and methods

### Plant growth and treatment for analysis

*Arabidopsis thaliana *seeds of the Columbia ecotype were surface-sterilized and plated on designated media and vernalized for 48 h at 8°C. Plants were grown semi-hydroponically under 16-h-light (70 E/m^2^/sec)/8-h-dark cycles at a constant temperature of 23°C on basal Murashige and Skoog (MS) medium (Life Technologies) supplemented with 2 mM KNO_3_, 2 mM NH_4_NO_3 _and 30 mM sucrose [37]. Two-week-old seedlings were transferred to fresh MS media without nitrogen (KNO_3 _and NH_4_NO_3_) or carbon (sucrose) and dark-adapted for 48 h. To perform specific metabolic treatments, 25 dark-adapted seedlings were transferred to fresh MS medium containing 0% or 1% (w/v) sucrose and/or 2 mM KNO_3 _and 2 mM NH_4_NO_3 _or no nitrogen, and illuminated with white light for an additional 8 h (70 E/m^2^/s^1^). Following these transient carbon and nitrogen treatments, whole seedlings were harvested, immediately frozen in liquid nitrogen, and stored at -80°C before RNA extraction.

### RNA isolation and microarray analysis

RNA was isolated from whole seedlings using a phenol extraction protocol as previously described [38]. Double-stranded cDNA was synthesized from 8 μg total RNA using a T7-Oligo (dT) promoter primer and reagents recommended by Affymetrix. Biotin-labeled cRNA was synthesized using the Enzo BioArray High Yield RNA Transcript Labeling Kit. The concentration and quality of cRNA was estimated through an A260/280 nm reading and running 1:40 of a sample on a 1% (w/v) agarose gel. cRNA (15 μg) was used for hybridization (16 h at 42°C) to the *Arabidopsis *ATH1 Target (Affymetrix). Washing, staining and scanning were carried out as recommended by the Affymetrix instruction manual. Expression analysis was performed with the Affymetrix Microarray Suite software (version 5.0) set at default values with a target intensity set to 150. Three biological replicates for each treatment were carried out.

### Using Affymetrix probes to assign genes to InterAct classes

Only Affymetrix probes representing genes that were deemed to be expressed in all treatments and replicates were used for subsequent analysis by InterAct Class [13,20]. For a gene to be considered expressed, the absolute call made by Affymetrix Microarray Suite 5.0 must be 'present' (P) for each of three replicates for each of four treatments (12 chips total). These genes have reliable values assigned to them that can be used for further analysis, while the proper InterAct Class assignment of a gene with an A ('absent') call would not be ensured. It should also be noted that the always P genes are less noisy than the genes that have an A call (data not shown). In the InterAct Class analysis, four values were assigned to each gene on the basis of its response to carbon and/or nitrogen. The first three values are the expression due to carbon (the expression in treatment 2 minus the expression in treatment 1; see Figure 2), the expression due to nitrogen (the expression in treatment 2 minus the expression in treatment 1; see Figure 2), and the expression due to CN (the expression in treatment 4 minus the expression in treatment 1; see Figure 2). The fourth InterAct Class value represents the expected expression due to C+N, which was calculated by adding the expression due to carbon to the expression due to nitrogen. The expression due to carbon, the expression due to nitrogen, the expression due to CN and the C+N values were calculated for each replicate and then analyzed with InterAct Class without binning [20].

### Statistical analysis of InterAct Classes and functional categories

*p*-values were calculated for the MIPS functional categories (funcats) [21,22] analysis as described previously [13]. Briefly, the number of genes assigned to the funcat being analyzed and any InterAct class was used as *n*; *p *was the number of genes assigned to the specific model being analyzed divided by the number of genes assigned to an InterAct class and funcat; *k *was the number of genes in the funcat being analyzed and assigned to the model being analyzed. This analysis, with the baseline being all the genes assigned an InterAct class, accounts for any biases that may have been caused by discarding all the absent genes. The one-tailed *p*-value was considered when the Poisson approximation of binomial probabilities was used. For the binomial-ratio and the exact binomial probability test, the *p*-value for *k *or more out of *n *was used.

### Identification of putative *cis*-regulatory elements in promoters of CN-regulated genes

Pathways whose genes are over-represented in Model 3 CN-enhanced were identified using the informatic tool PathExplore [23] function 13 [24]; the methodology is described in pages at these websites. Briefly, a binomial test is used, and the genes assigned an InterAct class were used as the parent list, *n *was the number of genes in Model 3 CN-enhanced (the child list), *k *was the number of genes in the pathway being analyzed and in the child list, and *p *was the number of genes in the pathway being analyzed and in the parent list divided by the number of genes in the parent list. We limited our search to pathways that contained more than two genes in the Model 3 CN-enhanced list. To identify *cis*-regulatory elements involved in regulating genes in Model 3 CN-enhanced and protein synthesis, we used genes involved in protein synthesis that were assigned Model 3 CN-enhanced, to drive the *cis *search: At1g07070 (60S ribosomal protein L35a), At2g36620 (60S ribosomal protein L24), At5g07090 (ribosomal protein S4), and At5g58420 (ribosomal protein S4 like).

The methodology used to identify putative carbon and CN regulatory elements was carried out as described previously [11]. RSA tools was used to extract the *A. thaliana *promoters for every gene [39,40]; AlignAce was then used to identify over-represented motifs in the promoters of the genes being analyzed (AlignAce motifs) [24]. To determine if a motif is over-represented in the promoters of genes in a particular sub-model, the sequence extracted from RSA tools and its reverse complement were searched to determine how many promoters contained the AlignAce motif and in what copy number. Then a binomial test was used to determine if the number promoters that contain the motif in the proper number of copies are over-represented in a particular sub-model. For this analysis, the number of genes with the AlignAce motif being analyzed in their promoter is *n*, *p *is the number of genes in the sub-model (for example, Model 3 CN-enhanced) divided by the total number of genes assigned an InterAct class, and *k *is the number of genes whose promoters contain the AlignAce motif being analyzed (in a specific copy number) and that is in the particular sub-model being tested. A *p*-value was only calculated if *k *is greater than nine. In each case, the lowest *p*-value is given. *Cis *elements over-represented in the C-only inductive model are considered to be putative C-elements, and *cis *elements that are over-represented in the promoters of Model 3 CN-enhanced genes and are not over-represented in the promoters of C-only inductive genes, are considered to be putative CN-elements (Table 5).

To identify interacting elements, a similar analysis was used. For example, to identify motifs interacting with a C-element (Table 5) in regulating induction due to carbon (C-associated elements), genes whose promoters contain the C-element were identified. The promoters of these genes were then checked for a second motif. The number of genes that contained the C-element being analyzed and the second motif was used as *n*. The number of genes in the C-only inductive model that contained the C-element being analyzed divided by the number of genes assigned an InterAct class and that contained the C-element being analyzed was used as *p*. The number of genes whose promoters contain that C-element and the second motif being analyzed (in a specified copy number) and that are in the C-only inductive model was used as *k*. In this example, the analysis will determine if the genes that contain the second motif and the C-element being analyzed are over-represented in the C-only inductive model compared to the genes that just contain the C-element. The same approach was used to identify NDEs as described below.

### Further analysis for NDEs

The 33 motifs (13 motifs from ribosomal proteins plus 20 motifs from ferredoxin-related proteins (data not shown)) that are not N-, CN- or C-associated elements were tested to determine whether they are potential NDEs. They were tested to see whether genes whose promoters contained these motifs plus a C-element (Table 5) are over-represented in Model 3 CN-enhanced, as compared to all the genes whose promoters contain the C-element as described above. If a *p*-value less than 0.05 is obtained, the C-element and potential NDE are a significant combination and are likely to regulate carbon and nitrogen interactions. As each motif is tested with each of the 11 C-elements, two steps were taken to control for the multiple tests. First, single strands of the promoter sequences of the *A. thaliana *genes were randomized 200 times, the reverse complement of the randomized strand was determined, and the number of times the 33 remaining AlignAce motifs were found to be over-represented (*p*-value < 0.05) with the C-elements was determined and compared to the number of significant combinations (*p*-value < 0.05) between the 33 remaining motifs and the C-elements when the actual promoters were used. In no set of the randomized promoters were the potential NDEs found to form more significant combinations with the 11 C-elements than the actual promoter sequences (*p*-value < 1/200 = 0.005). In the second control step, the number of significant combinations that each of the 33 remaining AlignAce motifs was involved in was determined and compared to the number of significant combinations found with the 200 sets of randomized promoters. For one motif, if one random set is significant with as many C-elements as the real promoters the *p*-value would be 0.005 (1/200).

### Further analysis of CN1 and CN2

The promoter for At2g15620 was extracted from RSA tools [39,40]. The reverse complement of the strand from RSA tools was determined to identify the occurrence of CN1 and CN2 in either strand of the promoter as described above to determine over-representation of the AlignAce motifs in the promoters of the genes in Model 3 CN-enhanced. To determine whether CN1 and CN2 occur more times than expected in the promoter, the sequence from RSA tools [39,40] was randomized 5,000 times and the above procedure was repeated. The number of times CN1 and/or CN2 were found in the randomized versions as many or more times than the actual promoter was determined and used to calculate a *p*-value (that is, if 50 random cases do as well as or better than the actual case *p*-value = 50/5,000 (0.05))

The sequence database was searched using BLAST [29] for a gene similar to At2g15620 in the *O. sativa *sequence. Only one hit was found. This gene is annotated as a ferredoxin-dependent nitrate reductase [28]. The 1,000 base-pairs upstream of this gene were taken and 'BLAST align two sequences' was used to determine whether this sequence is similar to the promoter of At2g15620. BLAST did not find enough similarity to create an alignment. The sequence was then subjected to the same test described above for the promoter of At2g15620.

### Funcat analysis of the NDEs

Funcat analysis of the genes whose promoters contain specific *cis *elements was performed similarly to the approach described above. Briefly, the number of genes assigned to the funcat being analyzed and Model 3 CN-enhanced was used as *n*; *p *was the number of genes assigned to Model 3 CN-enhanced and the funcat being analyzed divided by the number of genes assigned to Model 3 CN-enhanced and a funcat; *k *was the number of genes in the funcat being analyzed that was assigned to the Model 3 CN-enhanced category and containing the combination of C- and N-element being analyzed.

Statistical significance of localization was calculated similarly. The only difference being that instead of genes assigned a funcat, genes whose protein products are predicted to be localized in the compartment being analyzed were used. Predicted protein localizations were extracted from the TAIR web page [25].

## Additional data files

The following additional data are available with the online version of this paper: Additional data file 1 containing a table listing the Affymetrix probe ID, gene, and InterAct class for all the Affymetrix probes assigned an InterAct class; Additional data file 2 listing the data from 12 Affymetrix microarray chips used in this study.

## Supplementary Material

Additional data file 1 A table listing the Affymetrix probe ID, gene, and InterAct class for all the Affymetrix probes assigned an InterAct class Click here for additional data file

Additional data file 2 The data from 12 Affymetrix microarray chips used in this study Click here for additional data file

## Figures and Tables

**Table 1:** InterAct classes that contain more than one gene

CN model		Number of genes	InterAct class			
			
			C	N	CN	C+N
No effect		1,167	0	0	0	0
			
C-only	Inductive	1,011	1	0	1	1
	Repressive	596	-1	0	-1	-1
			
N-only	Repressive	4	0	-1	-1	-1
	C dominates	187	1	-1	1	1
		156	-1	1	-1	-1
		142	2	1	2	2
		140	-2	-1	-2	-2
			
Model 1 (independent)	N dominates	3	-1	1	1	1
	Equal effect	145	-1	-1	-1	-1
		63	1	1	1	1
	Antagonistic	6	2	-1	1	1
		3	-2	1	-1	-1
	Inductive	4	2	1	3	3
		3	1	1	2	2
	Repressive	3	-1	-1	-2	-2
		2	-2	-1	-3	-3
			
			
Model 2 (dependent)	Inductive	7	0	0	1	0
	Repressive	2	0	0	-1	0
			
Model 3 (dependent/ independent)	CN-enhanced	92	1	0	2	1
		25	2	1	3	2
		13	-1	-1	-1	-2
		11	-2	-1	-2	-3
		8	2	-1	2	1
		8	1	-1	2	1
		4	0	-1	0	-1
		3	0	-1	1	-1
		2	2	1	4	3
		2	-3	-1	-2	-3
			
	CN-suppressed	46	-2	1	-2	-1
		17	-1	1	-1	0
		9	-1	2	-1	1
		9	-1	0	-2	-1
		9	1	1	1	2
		8	-1	1	-1	1
		6	0	1	0	1
		6	2	0	1	2
		3	2	1	2	3
		2	1	1	-1	1
		2	-1	1	0	1
		2	1	2	1	3
		2	1	0	0	1
		2	1	-1	-1	1
		2	0	1	-1	1

**Table 2:** Funcats that are statistically under-represented in InterAct class 0000 (the No effect model)

Funcats	Number of genes assigned an InterAct class	Number of InterAct class 0000 genes	*p*-value
All funcats	3,447	1,089	-
Metabolism	496	120	6.0 × 10^-4^
Protein synthesis	218	55	2.6 × 10^-2^
Energy	125	21	1.34 × 10^-4^

**Table 3:** Sub-models that are misrepresented in the metabolism, protein synthesis and energy funcats

Funcats	No effect (1,089)	C-only		CN interactions		
				
				Model 1	Model 3	
		
		Inductive (675)	Repressive (567)	Equal effect (195)	CN suppressed (127)	CN enhanced (163)
Metabolism	120 -S	141 +S	62 -S	19 -S	34 +S	20
Protein synthesis	55 -S	81 +S	13 -S	4 -S	2 -S	32 +S
Energy	21 -S	32	29 +S	3	8	5

+S, sub-model over-represented; -S, sub-model under-represented. See text for details.

**Table 4:** Genes used to drive *cis *analysis

Gene	Enzyme	Class			
		
		C	N	CN	C+N
**(a) **Genes from pathways that are over-represented in Model 3 CN enhanced					
At2g27510	Ferredoxin	1	0	2	1
At1g30510	Ferredoxin-NADP^+ ^reductase	1	0	2	1
At4g05390	Ferredoxin-NADP^+ ^reductase	2	1	3	2
					
**(b) **Genes involved in protein synthesis were also used to drive the *cis *analysis					
At1g07070	60S Ribosomal protein L35a	1	0	2	1
At2g36620	60S Ribosomal protein L24	1	0	2	1
At5g07090	40S Ribosomal protein S4	1	0	2	1
At5g58420	40S Ribosomal protein S4	1	0	2	1

**Table 5:** Motifs that are over-represented in Model 3 CN-enhanced or in the C-only inductive model

Motif	C-only inductive *p*-value	Model 3 CN-enhanced *p*-value	Element name
Ferredoxin-related motifs			
RGAAVMANA	NS	0.0262	CN1
GNAANVMGAHNM	NS	0.0089	CN2
GAWYTGA	0.0073	NS	C1
ARNNGANNCAA	0.00049	NS	C2
KMSAGAG	0.0322	NS	C3
WMNCHGAANC	0.0091	NS	C4
GAGARRDDG	0.0375	NS	C5
			
Protein-synthesis related motifs			
WKGGGCC	<0.0001	<0.0001	C6
GGCCSAW	<0.0001	<0.0001	C7
AAACYCNA	0.0375	0.0038	C8
WTBGGCY	0.0022	0.011	C9
GDNTTGKAM	0.0359	NS	C10
AAGAAAA	0.0344	NS	C11

Nucleotide abbreviations: R; A or G, Y; C or T, W; A or T, S; G or C, M; A or C, K; G or T, H; A, C or T, B; G, C or T, V; G, A or C, D; G, A or TC, N; G, A, C or T.

**Table 6:** Potential NDEs

NDEs	Element name	Ferredoxin C-elements	Protein synthesis C-elements			Total *p*-value
			
		KMSAGAG (C3)	WKGGGCC (C6)	GGCCSAW (C7)	GDNTTGKAM (C10)	
Ferredoxin-related motifs						
CHHNAACHRA	N1	NS	0.0222	0.0344	NS	0.046
			N1C6	N1C7		
						
Protein synthesis related motifs						
TNNDNVNACAACA	N2	0.0281	0.0207	0.0268	0.0037	<0.005
		N2C3	N2C6	N2C7	N2C8	

For nucleotide abbreviations see the foonote for Table 5.

**Table 7:** Misrepresentation of genes that are potentially regulated by a combination of a C-element and N1 or N2

Gene set	Protein synthesis funcat	Genes predicted to be localized to the mitochondria
InterAct Class genes (3,652)	370	393
Model 3 CN enhanced (127)	32 +S	21
N1C6 (45)	14 +S	9
N1C7 (49)	15 +S	9
N2C3 (27)	5	4
N2C6 (16)	8 +S	7 +S
N2C7 (17)	9 +S	7 +S
N2C10 (15)	3	4

+S, sub-model over-represented; -S, sub-model under-represented. See text for details.
